# The Effect of Long Lasting Insecticide Bed Net Use on Malaria Prevalence in the Tombel Health District, South West Region-Cameroon

**DOI:** 10.1155/2016/3216017

**Published:** 2016-06-15

**Authors:** Eric B. Fokam, Kevin T. J. Dzi, Leonard Ngimuh, Peter Enyong

**Affiliations:** ^1^Department of Zoology and Animal Physiology, University of Buea, P.O. Box 63, Buea, Cameroon; ^2^Department of Microbiology and Parasitology, University of Buea, P.O. Box 63, Buea, Cameroon

## Abstract

Malaria remains a major public health problem in Africa, and its prevalence in Cameroon stands at 29%. Long Lasting Insecticide Nets (LLINs) were distributed in 2011 to reduce malaria mortality and morbidity; however, assessment of this intervention is scanty. The present study in the Tombel health district (THD) investigated the impact of this distribution on malaria prevalence. A total of 31,657 hospital records from 3 health facilities in 3 health areas for 2010–2013 were examined. Records for 2010 and 2011 provided predistribution baseline data, while those of 2012 and 2013 represented postdistribution data. 8,679 (27.4%) patients were positive for malaria. Children below 5 years had the highest prevalence (40.7%). The number of confirmed cases was highest from June to August (peak rainy season). Malaria prevalence was higher in males (25.3%) than in females (23.2%). Malaria prevalence increased in THD from 26.7% in 2010 to 30.7% in 2011 but dropped to 22.7% in 2012 and then increased in 2013 to 29.5%. There was an overall drop in the total number of confirmed malaria cases in 2012; this decrease was significant in Ebonji (*p* < 0.001) and Nyasoso (*p* < 0.015) health areas. The distribution of LLINs led to a short lived reduction in malaria prevalence in THD. LLIN distribution and other control activities should be reinforced to keep malaria prevalence low especially among the 0–5-year group.

## 1. Background

Malaria is a life threatening disease caused by a parasite that is transmitted to humans by the female* Anopheles* mosquito [1]. It remains a major public health problem, particularly in Africa where over 90% of cases are recorded [2]. It is estimated that 214 million new cases and 438 000 deaths occur worldwide yearly. Most of those who die from malaria are children under the age of 5, and most of these children live in Sub-Saharan Africa [3]. In Africa, malaria accounts for an estimated 25% of all childhood mortality, excluding neonatal mortality [4].

Malaria can be prevented by taking antimalarial drugs and by controlling the vectors that transmit the disease. This can be done in many ways such as spraying larvicides in the development sites of mosquitoes to kill larvae; household residual spraying (IRS) of insecticides and repellents to prevent mosquito bites. Despite the efforts to combat malaria through vector control and treatment using antimalarial drugs, malaria remains a major problem in Sub-Saharan Africa primarily due to drug and insecticide resistance and socioeconomic underdevelopment. The Roll Back Malaria Strategy recommends a combination of interventions for malaria control.

WHO (2015) recommends vector control using insecticide treated bed nets (ITNs) as one of the best ways to prevent malaria. ITNs offer a double protection to those who sleep under nets and to persons who do not use nets by reducing the number of malaria infected mosquitoes in the community. Recently pyrethroid treated Long Lasting Insecticide Nets (LLINs) were produced, which can last for at least 4 years before replacement [5]. A LLIN is a factory-treated mosquito net made with netting material that has insecticide incorporated within or bound around the fibres. The net must retain its effective biological activity without retreatment for at least 20 WHO standard washes under laboratory conditions and three years of recommended use under field condition. LLINs have been associated with a sharp decrease in malaria in countries where malaria programmes have achieved high LLIN coverage [6]. Since adoption of ITNs as a preventive tool for malaria in 2002, several campaigns of free distribution of LLINs have been conducted all over the country, with priority given to pregnant women and children below five years. However, a study carried out in Cameroon in 2012 suggested that 59.7% of households possessed at least one net while only 42.6% of households slept under these nets [7]. The objective of the Ministry of Public Health was to have 80% of the population sleeping under LLINs by 2015.

In 2011, Cameroon and its health partners distributed over eight million LLINs in all the ten regions of the country through the various health districts in an effort to reduce the burden of malaria in the country [8]. The Tombel health district (THD) in Cameroon has abundant rainfall which entertains numerous mosquito larval developmental and adult resting sites. The quality of housing (mostly plank with no ceiling and screens on doors or windows) in the area exposes people to intense mosquito bites and makes them vulnerable to malaria attack. However, no information is available on the impact of the intervention (free distribution of LLINs) on malaria morbidity in this region, especially as the country has embarked on a second mass distribution campaign. This study therefore sought to investigate the effect of LLINs use on malaria prevalence in South West Cameroon using Tombel health district (THD) as a case study.

## 2. Materials and Methods

### 2.1. Study Area

THD found in the Kupe Muanenguba division is a cosmopolitan inhabited by people from different ethnic groups. It is made up of 7 health areas, Baseng, Ebonji, Nyasoso, Edibenjock, Ndom, Tombel, and Ndibenjock. Three of these health areas, Tombel, Nyasoso, and Ebonji, were selected for the study by purposive sampling because of the high population in these areas and also because only health facilities in these health areas had trained laboratory personnel to run laboratory tests. The laboratories here used microscopy for detecting malaria in patients. The climate is warm and humid with 2 main seasons, long rainy (8-9 months) and dry (3-4 months) seasons, which induce abundant vegetation. Inhabitants here live mostly in plank houses. The major activity in this area is cocoa and plantain farming.

### 2.2. Study Design

A retrospective study was carried out using hospital records obtained from the Tombel District Hospital, Presbyterian General Hospital Nyasoso, and Integrated Health Centre Ebonji for the period January 2010–December 2013.

### 2.3. Data Collection

Because of lack of data collected before the distribution of LLINs in the THD, records for the two years before the intervention were compared to those of two years after distribution. Before the start of the study, the study site was surveyed using our data quality assessment tools which tested mainly for completeness and accuracy of data; thus, the study involved only health facilities that met the inclusion criteria used that included the following:

1. Patient must have done microscopic test to confirm malaria.
2. The age and sex must be registered.
3. The data for the study period must be available.

A total of 31,657 patient records were consulted from the 3 health centers, and information on the number of inpatients and outpatients confirmed for malaria was retrieved for the pre- (2010 and 2011) and postdistribution (2012 and 2013) periods of LLINs.

### 2.4. Data Analysis

Data collected was entered and analyzed using SPSS version 20. The Kruskal-Wallis test and chi square test were used to determine and compare malaria prevalence where necessary and a *p* value < 0.05 was considered statistically significant.

### 2.5. Ethics Statement

This study was approved by the Regional Delegation of Public Health Southwest and the Faculty of Health Science Institutional Review Board, University of Buea, Cameroon. Written permission was also obtained from the authorities of the various health centers where the study took place.

## 3. Results

### 3.1. Characteristics of Study Population

The records of 31,657 patients were consulted for malaria in the THD, of which 18,153 were males and 13,504 were females. Children less than five had the highest number of inpatient (48.8%) and outpatient (38.3%) malaria cases. The highest number of consultations (9,462) was registered between September and November.

### 3.2. Overall Malaria Prevalence

The overall prevalence of malaria in the THD for the study period was 27.4%. The prevalence of malaria in the THD for the predistribution period was 26.7% in 2010 and 30.7% in 2011. This was higher than that for the postdistribution period of 2012, which was 22.7%, indicating a decrease in malaria prevalence in the year 2012. However, there was an increase in the prevalence of malaria in 2013 to 29.5%.

The number of confirmed malaria cases was different across the years (Table 1), with 2012 having the lowest number of malaria cases for all health areas.

### 3.3. Malaria Prevalence in the Different Categories

Children below 5 years had highest malaria prevalence (40.7%), followed by persons greater than 5 years with malaria prevalence of 22.5%. Malaria prevalence in pregnant women was 20.3%. Malaria infection was statistically associated with the 3 different categories of persons (children < 5 yrs, persons > 5 yrs, and pregnant women) (Table 2).

### 3.4. Malaria Prevalence with Respect to Age

Malaria prevalence was higher in males (25.37%) than in females (23.37%) during the study period (Table 3) but the difference was not statistically significant.

### 3.5. Seasonality of Malaria Prevalence

In relation to seasonal transmission of malaria, the months of June–August popularly known as the rainy season in Cameroon had the highest percentage of malaria cases (32.7%). Malaria infection was significantly associated with season (Table 4).

## 4. Discussion, Conclusions, Recommendations, and Limitations

### 4.1. Discussion

Malaria is a major public health problem in Cameroon and is responsible for 31% of all consultations and 44% of all hospitalizations in health facilities [9]. The health centers chosen for the study used microscopy for diagnosing malaria in their laboratories which is the gold standard set by WHO for malaria diagnosis.

The present study has revealed malaria prevalence of 27.4% during the study period 2010–2013 in the THD. This result is slightly lower than the reported national value of 29% [10]. This result shows that hospital records could be indicative of malaria prevalence in the country since prevalence rates tie almost with those of other studies in Cameroon, which used blood smears. However, the malaria prevalence in patients observed in the study was 12% lower than that reported in a similar study conducted in Ethiopia by Alemu et al. [11].

The prevalence of malaria in inpatients in the THD during the postdistribution period (13.7%) was lower than that of the predistribution period (19.4%) suggesting that the distribution of LLINs had an impact in reducing malaria prevalence in the THD.

The prevalence of malaria in inpatients in the THD (33.10%) was also higher than the 29.80% reported by Ndong et al. [12], who carried out a similar study in the Northwest region of Cameroon, and also higher than that reported by a similar study conducted in Ethiopia by Ferede et al. [13], where the malaria prevalence was 17%. One of the reasons could be the fact that the climate of Tombel is warm and humid and favours growth of mosquitoes which constantly bite inhabitants as they sleep in their plank houses that most often have cracks in the walls and lack a ceiling that would serve as an additional barrier to protect them from infective bites.

The prevalence of malaria obtained from records of the selected period (2010–2013) was highest in children < 5 years (40.7%) followed by the persons > 5 years category (22.5%). The prevalence of malaria obtained from records of the selected period (2010–2013) for the children < 5 years old category was higher than that reported by Mbu et al. [14] in a study in Benin in which the prevalence of malaria in the under-5 age group was 22.4%.

The reduction in number of malaria cases in both inpatients and outpatients in the less-than-5 age group in the year 2012 (postdistribution period) suggests that the use of LLINs as one of the prevention strategies of malaria may have contributed in reducing the number of confirmed malaria cases. These results tie with results of Tokponnon et al. [15] who said that, after a nationwide campaign done in Kenya by the National Malaria Control Centre, where over 6 million bed nets were distributed between 2007 and 2009 to over a million households, there was a reduction in malaria parasitemia in children less than 5 years from 22% in 2006 to 16% in 2009.

In pregnant women, a prevalence of 20.3% was observed during the study period. This is lower than that provided by previous studies in Cameroon by Okiro and Snow [16] which indicated that 30% of pregnant women develop clinical malaria in Cameroon. Also, a study carried out by Tonga et al. [17] in the Sanaga Maritime division showed that the prevalence of malaria in pregnant women is 22.9%, which is still higher than that we got from our study. A possible explanation for the reduced malaria prevalence in pregnant women in the THD is the introduction of the intermittent preventive treatment (IPT) in combination with LLINs which have been shown to reduce malaria prevalence [18].

In all the health areas, there was a drop in the number of confirmed malaria cases in 2012 (postdistribution period) compared to the number of confirmed malaria cases in the predistribution period (2010, 2011). One of the reasons for the drop in prevalence and number of confirmed malaria cases in 2012 in the THD could be that LLINs had just been distributed in the THD from December 2011 to January 2012 and were still in use by many households since a lot of sensitization on bed net usage was going on. A decline in malaria burden attributed to the use of interventions such as ITNs and LLINs has also been reported in malaria-endemic countries such as Kenya [19] and Tanzania [20].

However, the drop in the number of confirmed malaria cases in 2012 was just temporary as in 2013. There was an increase in prevalence of malaria in both inpatients and outpatients in all health areas. This increase may be due to the fact that the population were excited and used nets for the first few months and then relaxed.

In the study area, malaria was observed in almost every month of the year, but there was significant difference in number of malaria cases across seasons (*p* < 0.05). Highest number of malaria cases were reported during the rainy season (June–August) followed by the early rainy season (March to May). The rainy season in Cameroon has been reported in other studies to be the season with highest malaria transmission rates in Cameroon [21].

The prevalence of malaria in both males and females was lower during the postdistribution period of LLINs compared to the predistribution period suggesting that LLINs distribution had an impact on malaria prevalence. However, the malaria prevalence was higher in males than in females for the study period but the difference was not statistically significant. A possible reason for this could be the fact that both males and females are involved in farming of cocoa and both parties are always present in the farm until late in the evening when malaria vectors become active. In addition, lots of domestic activities are undertaken outdoors in the evening (preparation of meals, drinking palm wine, relaxation, etc.), further exposing people to mosquito bites. Generally some confounding factors such as free treatment of malaria in children less than five, free distribution of IPT to pregnant women with its moderately high usage rate [22], change of national policy to use artemisinin combination therapy, and use of other mosquito control measures may have affected the results. In addition, since this work was based on retrospective hospital exploitation of records, a future follow-up community study should be conducted to assess the impact of the distribution and use of these LLINs with particular attention given to all confounders.

### 4.2. Conclusions

We can thus say that hospital records can be a good source of data for predicting malaria prevalence. A slight reduction was observed in the prevalence of malaria in 2012. The LLIN distribution campaign reduced malaria prevalence in the THD and thus LLINs distribution campaigns and other malaria control strategies should be sustained. The increase in malaria prevalence the following year after the distribution of nets tells us how continued education on use of nets is very necessary in the THD and probably elsewhere. Malaria prevalence was found to be significantly associated with the different categories of persons used in the study (children below 5 yrs, persons > 5 yrs, and pregnant women) and also with seasons.

### 4.3. Recommendations

- Proper education on the importance of utilising LLINs should be given to inhabitants of the THD by health personnel and community health workers.
- Another LLIN distribution campaign should be sustained by the Cameroon government since the LLINs that were distributed in 2011 will be worn out and ineffective in preventing malaria by 2015.

### 4.4. Limitation of the Study

We cannot attribute the reduction in malaria prevalence in the THD in 2012 solely to the distribution of LLINs in 2011, because other control measures such intermittent preventive treatment for pregnant women could have also contributed in reducing malaria morbidity in the THD, and our study design could not capture the contribution of each intervention.

## Figures and Tables

**Table 1:** Total number of confirmed malaria cases in the THD.

	Predistribution		Postdistribution		*p* value
	2010 (%)	2011 (%)	2012 (%)	2013 (%)	
Health area					
Ebonji	273 (31)	224 (25.4)	128 (14.5)	257 (29.1)	*p* < 0.05
Nyasoso	510 (29.5)	483 (28)	304 (17.6)	431 (24.9)	*p* < 0.05
Tombel	1638 (27)	1717 (28.3)	1205 (19.9)	1509 (24.9)	*p* > 0.05
THD	2421 (27.9)	2424 (27.9)	1637 (18.9)	2197 (25.3)	*p* > 0.05

**Table 2:** Prevalence of malaria in children < 5 years, persons > 5 years, and pregnant women in the THD from 2010 to 2013.

Category of persons	Inpatients (%) with malaria Predistribution period (2010/2011)	Inpatients (%) with malaria Postdistribution period (2010/2011)	Total number of inpatients (%) with malaria (2010/2011)	Out patients (%) with malaria Predistribution period (2010/2011)	Out patients (%) with malaria Postdistribution period (2010/2011)	Total number of outpatients (%) with malaria (2010/2011)	Overall prevalence (%) in the different categories of persons (2010–2013)	*p* value
CH < 5	26.5	22.3	48.8	18.5	19.8	38.3	40.7	*p* < 0.001
Persons > 5	16.6	10.1	26.7	12.4	10.0	21.4	22.5	
Pregnant women	22.2	19.4	41.6	8.3	1.6	9.9	20.3	
Tombel HD	19.4	13.7	33.1	14.8	12.1	25.9	27.4	

**Table 3:** Malaria prevalence in relation to sex in the THD during the study period (2010–2013).

	Predistribution period		Postdistribution period		*p* value
	2010 (%)	2011 (%)	2012 (%)	2013 (%)	
Gender					
Males	1470 (28.0)	1520 (29.0)	1234 (23.5)	1025 (19.5)	*p* > 0.05
Females	878 (25.6)	926 (27.0)	729 (21.3)	897 (26.1)	
THD	2348 (27.1)	2446 (28.1)	1963 (22.6)	1922 (22.1)	

**Table 4:** Seasonal variation of malaria prevalence in the THD during the study period (2010–2013).

Season	Number screened for malaria	Number confirmed positive	Percentage	*p* value
September–November	9467	2402	25.37	*p* < 0.001
December–February	8568	1993	23.26	
March–May	6556	1971	30.06	
June–August	7066	2313	32.73	
